# Transcriptomic and genetic evidence highlights EHHADH in a FUNDC1-associated mitochondrial network in diabetic nephropathy

**DOI:** 10.1016/j.metop.2026.100492

**Published:** 2026-08-13

**Authors:** Yuzhi Chen, Demei Ying, Xuli Guo, Shaozhe Wang, Wenjing Liu, Siwen Wang, Na Kuang, Jiahan Li, Nan Chen

**Affiliations:** a Hebei Key Laboratory of Medical Data Science, School of Medicine, Hebei University of Engineering, Handan, Hebei Province, 056038, China; b Department of Obstetrics and Gynecology, Xinqiao Hospital, Second Clinical Medical College of Army Medical University, Chongqing, 400037, China; c Department of Pathology, The First Affiliated Hospital of Hebei University of Chinese Medicine, Shijiazhuang, 050011, China

**Keywords:** FUNDC1, Diabetic nephropathy, EHHADH, Mitochondrial dysfunction, Mendelian randomization

## Abstract

**Background:**

Diabetic nephropathy (DN) is a major microvascular complication of diabetes and the leading cause of end-stage renal disease. Glomerular and tubular injury, mitochondrial dysfunction, and impaired mitophagy are core factors driving the progression of DN. Recent studies have identified FUNDC1 as a crucial mitophagy receptor; however, its regulatory mechanism in DN remains poorly elucidated.

**Methods:**

This study integrated compartmental transcriptomics, gene interaction network analysis, and summary-data-based Mendelian randomization (SMR) for systematic research. Differentially expressed genes (DEGs) were extracted from the glomerular dataset GSE96804 and the tubular dataset GSE294519. The intersecting genes of these DEGs with FUNDC1-interacting genes and mitochondrial dysfunction-related genes were obtained to screen core hub genes. Combined with renal expression quantitative trait locus (eQTL) data and DN genome-wide association study (GWAS) data, SMR analysis was performed. Functional enrichment analysis, gene set enrichment analysis (GSEA), and upstream transcription factor prediction were further conducted.

**Results:**

A total of 5560 DEGs were screened based on adjusted P values (Padj), including 2625 glomerular DEGs, 3199 tubular DEGs, and 264 common DEGs. Twenty FUNDC1-related core hub genes were finally identified, which were mainly enriched in mitophagy and mitochondrial energy metabolism pathways. After correction and screening via the HEIDI test, Transcriptomic and genetic evidence highlights EHHADH in a FUNDC1-associated mitochondrial network in diabetic nephropathy, and its expression was downregulated in DN lesional tissues. FUNDC1 and EHHADH were both associated with mitochondrial metabolic pathways, and E2F4 was predicted to be their common upstream regulatory factor.

**Conclusions:**

Multi-dimensional results suggest that EHHADH may represent a candidate gene within a FUNDC1-associated mitochondrial regulatory network in DN. The coordinated expression patterns of FUNDC1 and EHHADH suggest their potential involvement in mitochondrial homeostasis-related processes during DN progression.

## 1 Background

Diabetic nephropathy (DN) is one of the most common microvascular complications of diabetes and a leading cause of end-stage renal disease (ESRD). Its typical pathological manifestations include glomerulosclerosis, tubulointerstitial fibrosis, and impaired tubular epithelial function [1]. In recent years, research perspectives have gradually shifted from the isolated “glomerulus-centered” or “tubule-centered” single model to a holistic pathogenic framework, which highlights the synergistic injury between glomerular podocytes and renal tubular epithelial cells throughout DN progression. Cumulative evidence indicates that early podocyte lesions initiate downstream tubular metabolic stress, while progressive tubular fibrosis further aggravates glomerular filtration dysfunction; structural and functional defects of both renal compartments jointly determine disease prognosis [2,3].

At the mitochondrial functional level, both glomerular podocytes and proximal tubule epithelial cells depend on intact mitochondrial homeostasis to sustain their specialized physiological functions, yet they display distinct susceptibility to diabetic lipotoxicity and hypoxia [4]. Prior investigations have frequently observed disrupted mitochondrial quality control, endoplasmic reticulum stress and aberrant mitochondrial apoptotic signaling in both injured glomeruli and tubules of DN patients [5,6]. As core organelles governing cellular energy metabolism and cell survival, mitochondria rely on precise quality control systems to maintain cellular balance. Mitophagy, a subtype of selective autophagy responsible for eliminating damaged mitochondria, may serve as an important shared regulatory module across renal compartments, and its abnormal activity is speculated to participate in the mutual exacerbation of glomerular and tubular lesions in DN [7].

FUN14 domain-containing protein 1 (FUNDC1) is an outer mitochondrial membrane mitophagy receptor, which initiates LC3-dependent mitochondrial clearance under hypoxic and metabolic stress conditions [8]. Existing preclinical and clinical studies have implicated FUNDC1-mediated mitophagy in the progression of diabetic microvascular complications and acute renal injury [9,10]. In acute kidney injury models, FUNDC1 activation appears to relieve tubular oxidative stress and epithelial apoptosis [11]. Nevertheless, whether FUNDC1 participates in the synergistic pathogenic cascade of chronic DN, and how its regulatory network differs or coordinates between glomerular and tubular compartments, remains largely unclear.

From the analytical perspective, transcriptome profiling combined with network pharmacology can systematically decode complex gene interaction networks, which has been widely adopted to screen potential pathogenic hub molecules. Summary-data-based Mendelian randomization (SMR), a genetic association analysis tool, can partially mitigate confounding bias and reverse causality inherent to observational tissue research, offering suggestive population-level evidence for gene–disease associations [12]. In the present work, we set gene expression levels as exposure and DN disease susceptibility as outcome to infer potential genetic correlations. The combinatorial analytical strategy of compartmental transcriptomics, SMR and network pharmacology enables multi-dimensional exploration of mitochondrial regulatory signatures from genetic causality, molecular interaction and tissue-specific pathway layers.

**Fig. 1:**
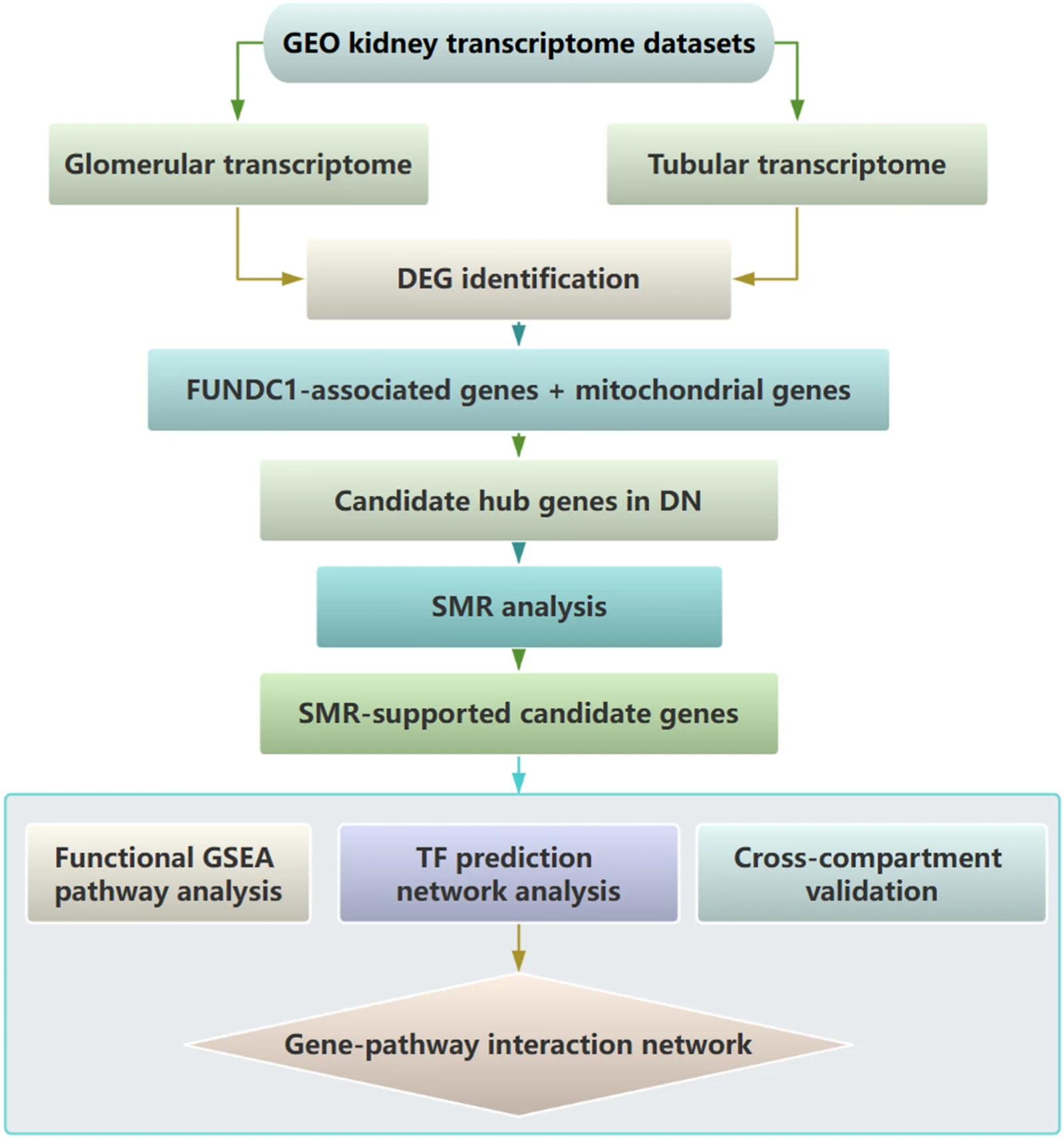
Research design.

Based on the above background, we integrated independent glomerular bulk transcriptome and high-purity spatial tubular transcriptomic cohorts, combined with SMR genetic inference and network pharmacology, to characterize the potential regulatory role of FUNDC1 in DN-associated mitochondrial impairment. We further aimed to screen key hub molecules mediating FUNDC1-related mitochondrial homeostasis, and explore the shared and compartment-specific transcriptional patterns of this mitophagy-lipid metabolic axis across glomerular and tubular compartments. This work may provide preliminary mechanistic clues explaining the coordinated progression of glomerular and tubular injury in DN, and uncover candidate genes with suggestive genetic correlation and prospective therapeutic value (Fig. 1).

## 2 Methods

### 2.1 Data sources

Gene expression data used in this study were downloaded from the Gene Expression Omnibus (GEO) database. Two datasets were included: the glomerular microarray dataset GSE96804, containing 41 biopsy-confirmed diabetic nephropathy (DN) samples and 20 healthy controls, and the tubular transcriptomic dataset GSE294519, consisting of 23 biopsy-confirmed DN samples and 13 healthy controls. All patients in both datasets were diagnosed by renal biopsy. Differential expression analysis was performed independently for each dataset, after which the DEGs identified from the two renal compartments were integrated to construct a comprehensive DN-related gene set. To construct the FUNDC1 interaction network, key interacting genes were retrieved from BioGRID, STRING, and GeneMANIA using default parameters. Mitochondrial dysfunction–related genes (MDRGs) were obtained from the GeneCards database by querying the keywords “mitochondrial dysfunction” and “mitophagy,” with a relevance score >5 as the selection criterion.

To evaluate the genetic relevance of candidate genes, cis-eQTL data of whole blood were obtained from eQTLGen (n = 31,684, predominantly of European ancestry), and DN GWAS summary statistics were retrieved from the GWAS Catalog (ID: GCST90475670; 25,234 DN cases and 413,872 controls). SMR analyses were performed using these datasets. All summary-level data were derived from European populations, and exposure and outcome datasets originated from independent GWAS consortia to minimize sample overlap and population heterogeneity.

### 2.2 Differential expression analysis

Differentially expressed genes (DEGs) were independently identified from the glomerular dataset GSE96804 and the tubular dataset GSE294519. For GSE96804, when multiple probes corresponded to the same gene symbol, the probe with the highest average expression was retained. Differential expression analysis was performed using empirical Bayes moderation, and P values were adjusted using the Benjamini–Hochberg method. Genes with an adjusted P value (false discovery rate, FDR) < 0.05 and an absolute log_2_ fold change (|log_2_FC|) > 0.5 were considered differentially expressed. Volcano plots and heatmaps were generated for visualization.

### 2.3 Identification of differential interaction genes and enrichment analysis

To identify FUNDC1-associated dysregulated modules in DN, DEGs, MDRGs, and FUNDC1-interacting genes were intersected to obtain FUNDC1–DN hub genes. Gene Ontology (GO) and Kyoto Encyclopedia of Genes and Genomes (KEGG) pathway enrichment analyses were subsequently performed.

### 2.4 SMR identification of key genes and functional characterization of candidate genes

Cis-eQTL variants with P < 5 × 10^−8^ were selected as instrumental variables and harmonized with DN GWAS summary statistics. Linkage disequilibrium pruning was performed using the 1000 Genomes Project reference panel (r^2^ < 0.001 within a 1000-kb window), and weak instruments (F-statistic < 10) were excluded. SMR analysis was conducted to evaluate the association between genetically predicted gene expression and DN. The HEIDI (heterogeneity in dependent instruments) test was subsequently performed to distinguish pleiotropy from linkage disequilibrium, and genes with SMR P < 0.05 and HEIDI P > 0.05 were considered candidate genes. For significant genes, regional association plots integrating cis-eQTL, GWAS, and SMR results were generated to visualize the local association signals.

For functional exploration, KEGG gene sets (c2.cp.kegg.v2026.1.Hs.symbols.gmt) were downloaded from MSigDB. Spearman correlation coefficients between each key gene and all other genes were calculated in the DN dataset, and genes were ranked according to the correlation coefficients. Gene set enrichment analysis (GSEA) was performed using the ranked gene list, with adjusted P < 0.05 and |NES| > 1 considered statistically significant. Based on the top enriched KEGG pathways, a FUNDC1–hub gene–pathway interaction network was constructed and visualized using Cytoscape. Correlation analysis between key genes was performed using |cor| > 0.3 and P < 0.05 as the significance criteria.

### 2.5 Construction of the transcriptional regulatory network and cross-compartment transcriptomic assessment

Potential upstream transcription factors (TFs) associated with key genes were predicted using ChEA3, which integrates multiple ChIP-seq–based resources. TFs were prioritized based on integrated enrichment scores. Top-ranked TFs were used to construct a TF–mRNA regulatory network and visualized.

To further evaluate the expression patterns of the final candidate genes across different renal compartments, the expression of the identified hub genes was additionally assessed in an independent transcriptomic dataset derived from a complementary renal compartment. The same differential expression analysis pipeline and statistical criteria described above were applied to evaluate the consistency of expression direction and statistical significance across renal tissue compartments.

### 2.6 Statistical analysis and software

All analyses were performed using R (version 4.2.3), SMR (version 1.3.1), and Cytoscape (version 3.10.3). Differential expression analysis of the microarray dataset was performed using the limma package. Data visualization was conducted using the ggplot2 and pheatmap packages. Functional enrichment analyses were performed using the clusterProfiler package, and correlation analyses were conducted using the Hmisc package. Multiple testing correction was performed using the Benjamini–Hochberg method.

## 3 Results

### 3.1 Identification of FUNDC1-associated DN hub genes

**Fig. 2:**
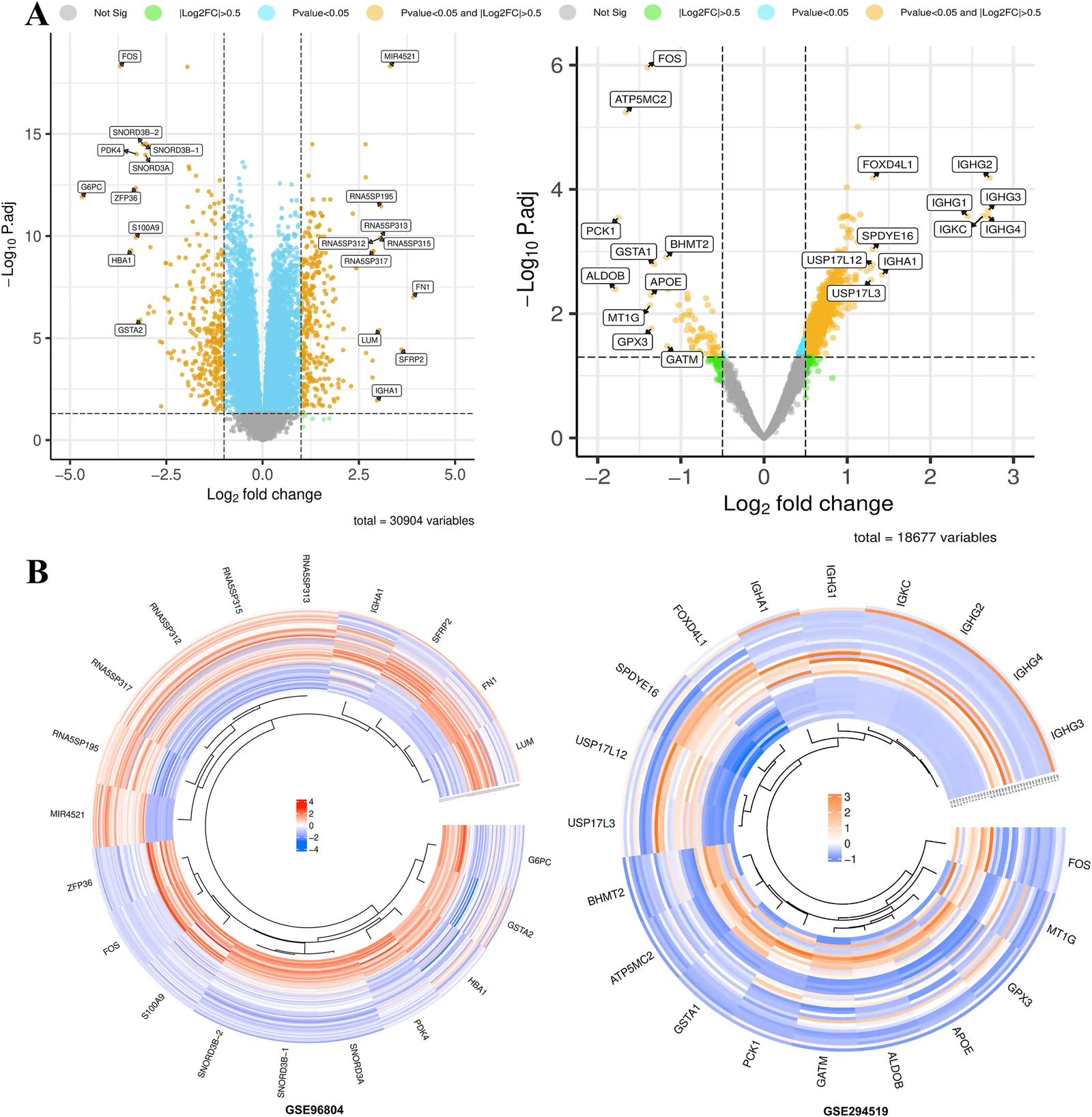
Volcano plot and heatmap for identifying differentially expressed genes (displaying the top 10 genes with upregulated or downregulated expression).

Transcriptomic analysis of the glomerular and tubular datasets identified a total of 5560 DEGs between DN and control groups. Specifically, 2625 DEGs were screened in glomerular tissues, including 1249 upregulated and 1376 downregulated genes, while 3199 DEGs were obtained in tubular tissues, consisting of 3119 upregulated and 80 downregulated genes (Supplementary Materials 1–2). Based on the ranking of |log_2_ fold change (FC)|, the top 10 upregulated genes (including NOD2, FOXM1, and CD53) and top 10 downregulated genes (including ACTA1, PRTG, and KIT) were selected and visualized via volcano plots and heatmaps (Fig. 2).

A total of 3611 mitochondrial dysfunction-related genes (MDRGs) were retrieved from the GeneCards database, and 130 FUNDC1-interacting genes were obtained from three gene interaction databases (Supplementary Materials 3–4). After intersecting the DEGs, MDRGs, and FUNDC1-interacting genes, 20 FUNDC1-associated hub genes potentially involved in DN were identified, including DHCR24, EHHADH, GABARAPL2, TUFM, BNIP3, FIS1, HIF1A, ITPR3, PPARGC1A, FUNDC1, USP30, TFEB, PACS2, TBK1, MIEF2, ATG5, MUL1, PRKN, ATG12, HSPA9.

### 3.2 Enrichment analysis

**Fig. 3:**
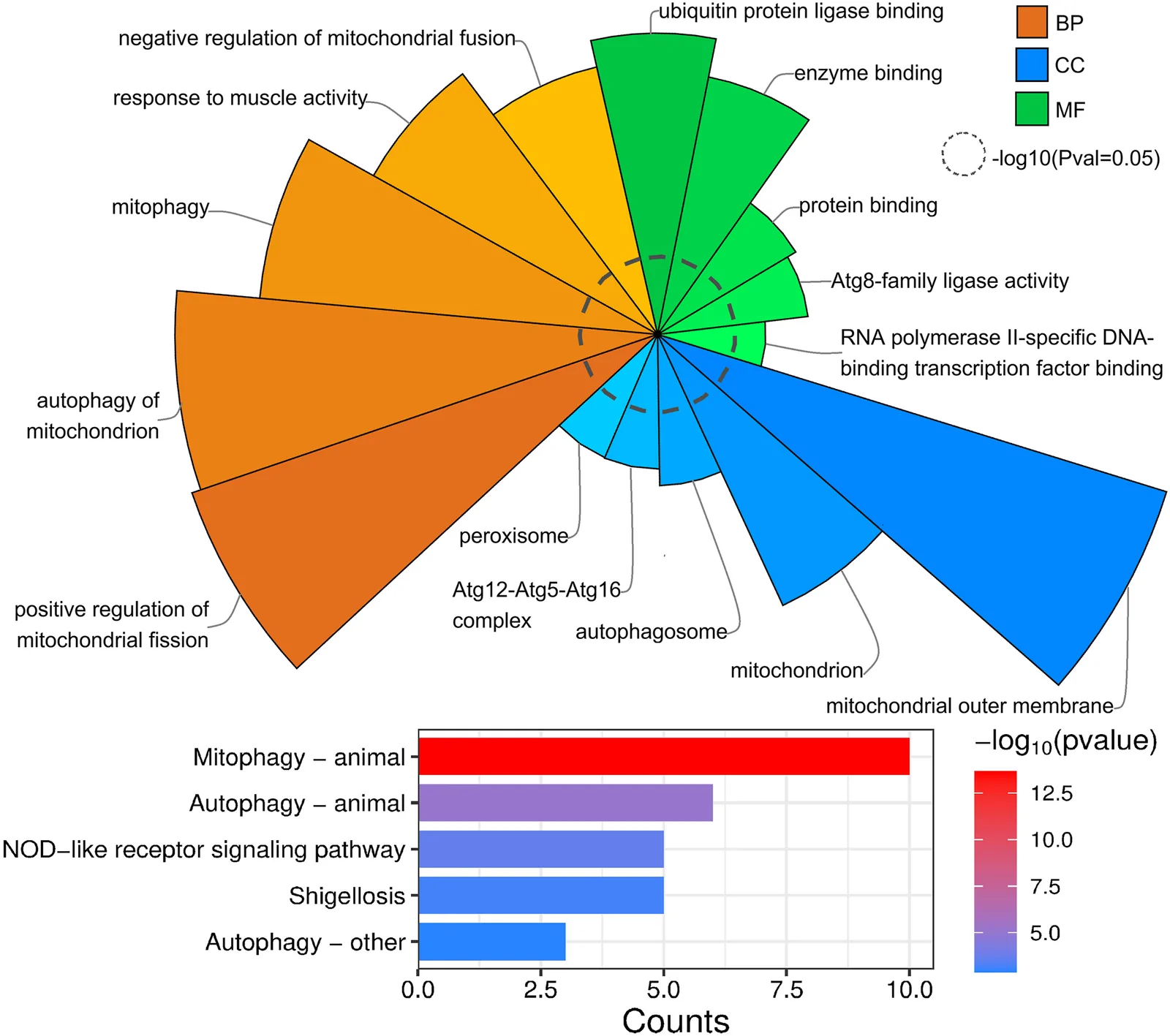
GO and KEGG enrichment analysis of FUNDC1–DN hub genes.

GO and KEGG enrichment analyses of the 20 hub genes identified 87 GO terms (61 BP, 18 CC, and 8 MF) and 8 KEGG pathways (Supplementary Material 5), which were primarily associated with mitophagy, mitochondrial organization, and hyperglycemia-related signaling pathways, suggesting that these genes may participate in pathways related to mitochondrial dysfunction and DN progression. (Fig. 3).

### 3.3 Identification of key genes by SMR analysis

**Fig. 4:**
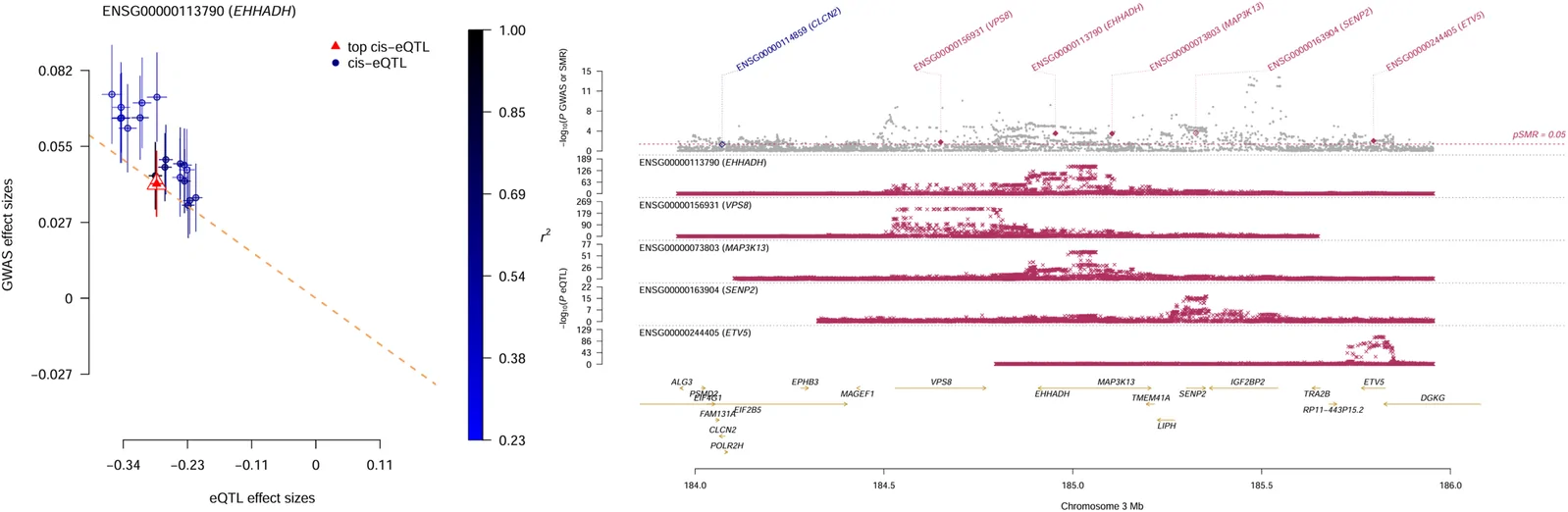
Results of SMR analysis of key gene.

SMR analysis of these hub genes initially identified two candidate genes (TUFM and EHHADH). After HEIDI testing, only EHHADH remained as a significant candidate gene associated with DN without evidence of heterogeneity (HEIDI P > 0.05). The effect estimate suggested an inverse genetic association between EHHADH expression and DN risk (β_SMR_ = −0.15), consistent with its downregulated expression pattern (log_2_FC < 0) (Supplementary Material 6). Genomic region analysis indicated that EHHADH is located within the 184.0–186.0 Mb interval on chromosome 3. In this region, eQTL, GWAS, and SMR signals overlapped, suggesting a potential shared genetic signal between EHHADH expression and DN susceptibility. These findings suggest that EHHADH may represent a candidate gene associated with DN in this locus and warrants further investigation (Fig. 4).

### 3.4 Functional and correlation analyses of key genes

Single-gene GSEA suggested that both FUNDC1 and EHHADH were predominantly enriched in mitochondrial-related processes, including mitochondrial matrix, mitochondrial protein complexes, and monocarboxylic acid catabolic processes (all P < 0.05, |NES| > 1). KEGG-based GSEA further revealed that FUNDC1 and EHHADH were significantly enriched in pathways related to respiratory chain complex I electron transport, thermogenesis, and SNCA/ABETA/PINK1 mutation-associated electron transport dysfunction (all P < 0.05, |NES| > 1) (Supplementary Material 7).

**Fig. 5:**
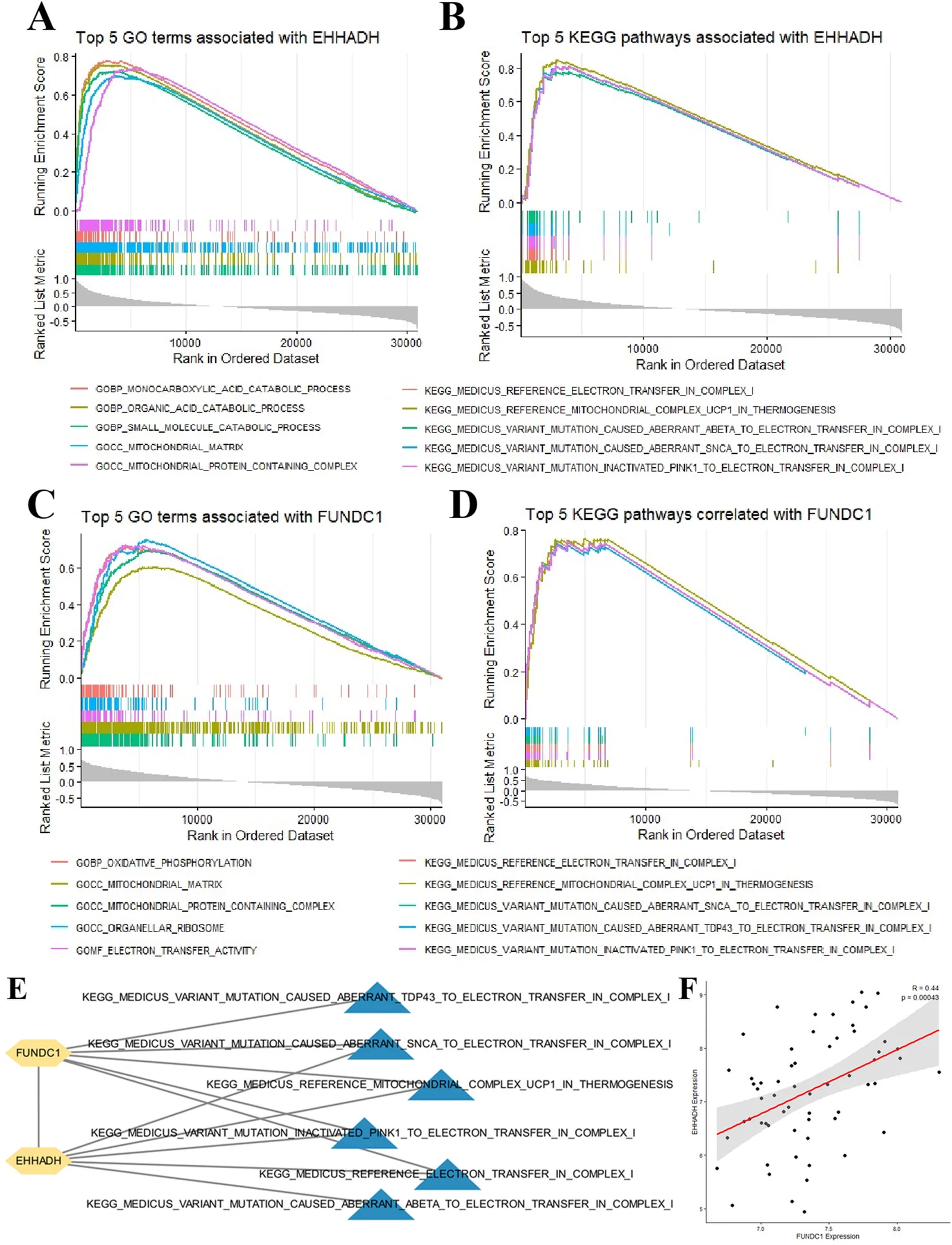
Functional and correlation analyses of key genes.

Based on FUNDC1, its hub gene EHHADH, and their top five enriched KEGG pathways, a FUNDC1–hub gene–pathway interaction network was constructed using Cytoscape. Correlation analysis showed a significant positive association between FUNDC1 and EHHADH expression (cor = 0.44, P = 0.00043), indicating a potential coordinated association in DN (Fig. 5).

### 3.5 TF regulatory network and cross-compartment transcriptomic assessment of hub gene expression

**Fig. 6:**
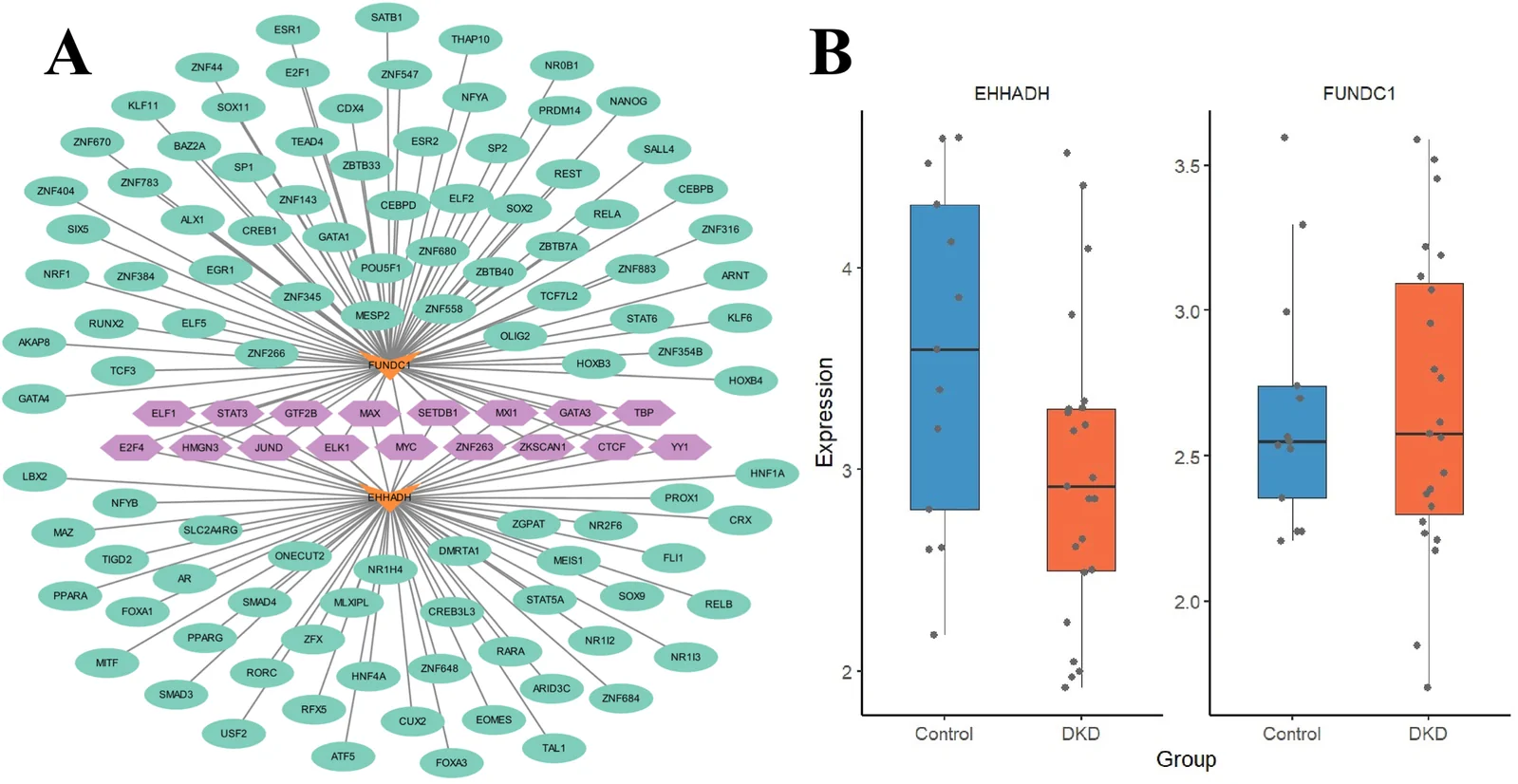
TF regulatory network of FUNDC1 and EHHADH and their tubular expression evaluation in DN.

TF prediction analysis identified 60 TFs regulating EHHADH and 77 TFs regulating FUNDC1, among which 17 TFs were shared (Supplementary Material 8). A TF–hub gene regulatory network was subsequently constructed, highlighting E2F4 as a candidate shared regulator of FUNDC1 and EHHADH (Fig. 6A).

To further evaluate the expression pattern of the identified hub genes across renal compartments, GSE294519 was analyzed using the same differential expression pipeline. Consistent with the direction observed in the discovery cohort, EHHADH remained downregulated in tubular tissues from patients with DN and showed nominal statistical significance (P = 0.0101), although the adjusted P value indicated borderline significance (FDR = 0.0549). In contrast, FUNDC1 expression did not differ significantly between DN and control samples (Fig. 6B–Supplementary Material 2).

## 4 Discussion

In this study, we integrated compartment-specific transcriptomic analysis, interaction network analysis, and SMR-based genetic inference to investigate the potential involvement of FUNDC1-associated mitochondrial regulatory networks in diabetic nephropathy (DN). Rather than establishing FUNDC1 as a direct causal driver, our analysis identified EHHADH as the key candidate gene within the FUNDC1-related network. EHHADH exhibited consistent downregulation across distinct renal compartments and was further supported by genetic association analysis, suggesting that altered EHHADH-associated metabolic regulation may represent a shared molecular feature associated with DN progression.

Mitochondrial dysfunction is increasingly recognized as a central pathological mechanism underlying DN development. Hyperglycemia-induced metabolic stress, excessive reactive oxygen species generation, and impaired mitochondrial quality control collectively contribute to progressive renal injury [13]. However, DN is not a homogeneous disease restricted to a single renal compartment. Increasing evidence indicates that disruption of the physiological interaction between glomerular and tubular compartments plays a critical role in disease progression. Podocyte injury can initiate downstream tubular metabolic stress, whereas tubular dysfunction further accelerates glomerular deterioration through inflammatory and metabolic feedback loops [14]. Therefore, identifying molecular regulators shared across different renal compartments may provide new insights into the coordinated progression of DN.

FUNDC1, an outer mitochondrial membrane mitophagy receptor, has been extensively implicated in mitochondrial quality control under metabolic and hypoxic stress conditions. Previous studies suggested that impaired FUNDC1-mediated mitophagy contributes to renal injury in diabetic models, partly through regulation of oxidative stress and mitochondrial apoptosis [15]. Consistent with these findings, FUNDC1-associated genes identified in our study were significantly enriched in mitochondrial organization, mitophagy, and energy metabolism pathways. However, our SMR analysis did not provide direct genetic evidence supporting FUNDC1 itself as a causal gene for DN. Therefore, FUNDC1 should be interpreted as an upstream molecular framework that guided the identification of mitochondrial regulatory candidates rather than as a genetically validated therapeutic target.

Among the FUNDC1-associated genes, EHHADH emerged as the primary candidate supported by both transcriptomic and genetic evidence. EHHADH encodes an enzyme involved in peroxisomal fatty acid β-oxidation and plays an essential role in maintaining lipid metabolic homeostasis in renal tubular epithelial cells. Previous experimental studies have suggested that EHHADH deficiency aggravates tubulointerstitial injury in diabetic kidney disease by disrupting peroxisomal function and promoting abnormal pexophagy, ultimately enhancing oxidative stress and inflammatory responses [16]. Our findings extend these observations by showing that reduced EHHADH expression is observed at the transcriptomic level and is further supported by SMR-based genetic association analysis.

The consistent reduction of EHHADH across glomerular and tubular compartments provides additional biological implications. Although renal compartments experience distinct pathological stresses during DN progression, including podocyte dysfunction in glomeruli and metabolic overload in tubular epithelial cells, both compartments are highly dependent on mitochondrial energy metabolism. The cross-compartment reduction of EHHADH suggests that impaired lipid metabolic regulation may represent a common molecular response during DN progression [17]. Importantly, while the biological functions of EHHADH have been primarily characterized in tubular cells, its downregulation in glomerular tissues indicates that EHHADH-associated metabolic disturbance may extend beyond tubular injury and participate in broader renal homeostasis disruption.

From a translational perspective, the SMR evidence obtained from whole-blood eQTL data provides additional significance. Although blood-derived genetic instruments cannot directly represent kidney-specific regulation, the identification of EHHADH as genetically associated with DN susceptibility suggests that its regulatory variation may reflect systemic biological processes linked to disease risk. Compared with purely tissue-level observational changes, genetic evidence is less susceptible to reverse causality and disease-induced secondary alterations [18]. Therefore, EHHADH may represent a potential molecular candidate associated with DN susceptibility., although kidney-specific eQTL and clinical cohort evaluation will be required to confirm its applicability.

Mechanistically, our pathway analyses indicated that FUNDC1 and EHHADH converge on mitochondrial metabolic regulation, suggesting a potential biological association between mitochondrial quality control and lipid metabolic regulation. EHHADH may primarily maintain metabolic substrate utilization through fatty acid oxidation, whereas FUNDC1 contributes to mitochondrial quality control through selective removal of damaged mitochondria. Similar coordinated regulation between different mitochondrial quality control processes has been reported previously, supporting the possibility that metabolic maintenance and mitochondrial clearance pathways may act cooperatively under stress conditions [19]. However, whether FUNDC1 directly regulates EHHADH expression or whether both genes represent parallel responses to diabetic stress remains unknown and requires experimental investigation.

Furthermore, transcription factor analysis identified 17 shared transcription factors potentially involved in the regulation of FUNDC1 and EHHADH. Among these candidates, E2F4 was prioritized based on integrated enrichment evidence and biological relevance. As a member of the E2F transcription factor family, E2F4 has been implicated in cellular stress responses, metabolic regulation, and renal pathological processes, including clear cell renal carcinoma [20,21]. Although diabetic nephropathy and renal cancer represent distinct diseases, they share certain pathological features, such as oxidative stress, inflammatory activation, and metabolic reprogramming. However, the current analysis does not establish a dominant role for E2F4 over other shared transcription factors; therefore, E2F4 should be considered a computationally prioritized candidate requiring further experimental validation.

Several limitations should be acknowledged. First, although SMR provides genetic evidence supporting an association between EHHADH expression and diabetic nephropathy risk, the eQTL dataset used in this study was derived from whole blood rather than kidney tissue. Therefore, kidney-specific regulatory effects require further confirmation using renal eQTL resources. Second, the present study was primarily based on transcriptomic integration and computational analyses, and experimental validation of the FUNDC1–EHHADH relationship, including functional perturbation, protein interaction, and histological confirmation, was not performed. Third, although cross-compartment transcriptomic analysis showed a consistent downregulation pattern of EHHADH, independent external cohorts with larger sample sizes are needed to further evaluate the robustness of these findings. Finally, the predicted transcriptional regulation by E2F4 and the proposed coordination between mitophagy and metabolic pathways remain hypothesis-generating and require further mechanistic studies.

Overall, this study identifies EHHADH as a genetically supported candidate gene within a FUNDC1-associated mitochondrial regulatory network in DN. These findings highlight the importance of metabolic-mitochondrial coupling in coordinating renal compartment injury and provide a potential molecular basis for future biomarker development and therapeutic exploration.

## 5 Conclusion

In this study, integrating transcriptomic analysis and SMR-based genetic association analysis, we identified a FUNDC1-associated mitochondrial regulatory network and prioritized EHHADH as a genetically supported candidate gene in DN-associated renal injury. Genetically predicted lower EHHADH expression was associated with increased DN risk, and both FUNDC1 and EHHADH were enriched in mitochondrial metabolic pathways, suggesting a potential association between mitochondrial quality control and fatty acid metabolism. Dysregulation of the FUNDC1–EHHADH-associated mitochondrial regulatory network may contribute to oxidative stress-related processes in DN, providing potential insights into mitochondrial homeostasis as a therapeutic consideration.

## CRediT authorship contribution statement

**Yuzhi Chen:** Conceptualization, Formal analysis, Funding acquisition, Methodology, Software, Supervision, Writing – original draft, Writing – review & editing. **Demei Ying:** Conceptualization, Project administration, Supervision, Validation, Writing – review & editing. **Xuli Guo:** Data curation, Funding acquisition, Methodology, Project administration, Resources. **Shaozhe Wang:** Data curation, Resources, Software, Supervision, Writing – original draft. **Wenjing Liu:** Investigation, Supervision, Validation. **Siwen Wang:** Investigation, Supervision, Validation. **Na Kuang:** Software, Visualization, Writing – review & editing. **Jiahan Li:** Formal analysis, Validation, Visualization. **Nan Chen:** Data curation, Formal analysis, Funding acquisition, Investigation, Methodology, Resources, Validation.

## Availability of data and materials

The GSE96804/GSE294519 transcriptomic dataset analyzed in this study is publicly available in the Gene Expression Omnibus (GEO) database under accession number GSE96804/GSE294519: https://www.ncbi.nlm.nih.gov/geo. Cis-eQTL summary statistics were obtained from the eQTLGen Consortium: https://www.eqtlgen.org. DN GWAS summary data were retrieved from the GWAS Catalog (accession: GCST90475670): https://www.ebi.ac.uk/gwas. Gene interaction data were derived from: BioGRID: https://thebiogrid.org; STRING: https://string-db.org; GeneMANIA: https://genemania.org. Mitochondrial dysfunction-related genes were collected from the GeneCards database: https://www.genecards.org. Potential upstream transcription factors were predicted using ChEA3: https://maayanlab.cloud/chea3. All datasets used in this study are publicly accessible. Processed data and supplementary materials generated during this study are included in the Supplementary Materials. Further information is available from the corresponding author upon reasonable request.

## Ethics approval and consent to participate

Not applicable: All data are from public databases and no additional ethical approval is required.

## Consent for publication

Not applicable: This study does not include any identifiable individual data or images requiring publication consent.

## Funding

This study was supported by Natural Science Foundation of Hebei Province (H2023402015), Science and Technology Research and Development Project of Handan City (21422083262); National College Students Innovation and Entrepreneurship Training Program (X202610076224; G202510076019) and Science Research Project of Hebei Education Department (QN2021032).

## Declaration of competing interests

The author team declares that there are no potential conflicts of interest.
